# A Nudge to the Truth: Atom Conservation as a Hard Constraint in Models of Atmospheric Composition Using a Species-Weighted Correction

**DOI:** 10.1021/acsestair.4c00220

**Published:** 2024-11-20

**Authors:** Patrick Obin Sturm, Sam J. Silva

**Affiliations:** † Department of Earth Sciences, University of Southern California, Los Angeles, California 90089, United States; ‡ Department of Environmental Engineering, University of Southern California, Los Angeles, California 90089, United States; § Department of Population and Public Health Sciences, University of Southern California, Los Angeles, California 90032, United States

**Keywords:** atmospheric chemistry, machine learning, hard constraints, mass conservation, weighted least-squares

## Abstract

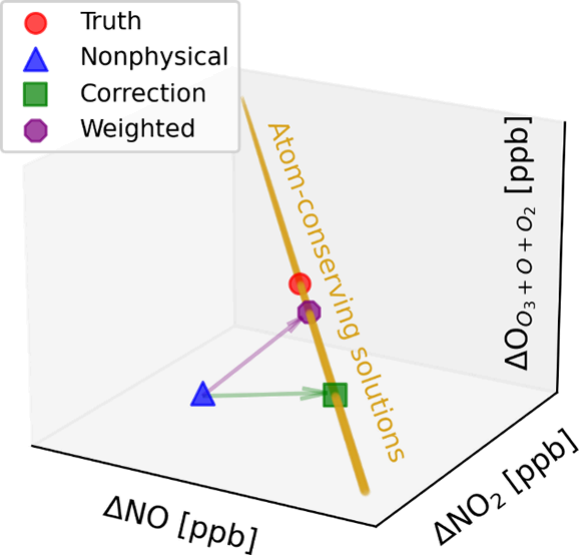

Computational models of atmospheric composition are not always physically consistent. For example, not all models respect fundamental conservation laws such as conservation of atoms in an interconnected chemical system. In well performing models, these unphysical deviations are often ignored because they are frequently minor, and thus only need a small nudge to perfectly conserve mass. Here we introduce a method that anchors a prediction from any numerical model to physically consistent hard constraints, nudging concentrations to the nearest solution that respects the conservation laws. This closed-form model-agnostic correction uses a single matrix operation to minimally perturb the predicted concentrations to ensure that atoms are conserved to machine precision. To demonstrate this approach, we train a gradient boosting decision tree ensemble to emulate a small reference model of ozone photochemistry and test the effect of the correction on accurate but nonconservative predictions. The nudging approach minimally perturbs the already well-predicted results for most species, but decreases the accuracy of important oxidants, including radicals. We develop a weighted extension of this nudging approach that considers the uncertainty and magnitude of each species in the correction. This species-level weighting approach is essential to accurately predict important low concentration species such as radicals. We find that applying the species-weighted correction slightly improves overall accuracy by nudging unphysical predictions to a more likely mass-conserving solution.

## Abstract 2

Computational models of atmospheric composition do not always make scientifically trustworthy predictions. This corrective approach minimally adjusts the predicted concentrations of chemical species to guarantee conservation of mass.

## 1 Introduction

Computational models of atmospheric chemistry are fundamental tools used to support basic research, inform environmental policies and assessments, and predict future atmospheric composition. However, these numerical models can sometimes violate core tenets of our understanding of the natural world, which includes the principle of conservation of mass, or atoms in the case of chemical reactions. This is often caused by parametrization choices, for example from incomplete reaction networks in chemical mechanisms,^1−3^ efficient numerical solvers,^4,5^ or transport in 3D air quality models.^6,7^ These violations are particularly common when using data-driven methods for parametrization which are not based solely on first-principles like many process-based models, but rather on a set of parameters optimized on some given data.

To address this problem, approaches for ensuring physical constraints in data-driven models have been developed for several applications in the Earth system sciences.^8−12^ For example, Geiss et al.^9^ constrain convolutional neural networks predicting the mixing ratios of atmospheric chemical species, to ensure the average of spatially super-resolved mixing ratios is consistent with the mixing ratio at their corresponding coarse grid cell, done analogously for conservation of energy or atmospheric water mass in Harder et al.^10^ Harder et al.^12^ address mass violation in aerosol fields by adjusting the variable with the least accurate predictions to satisfy mass conservation; this is related to a general approach proposed by Beucler et al.^8^ which constrains a subset of the output by the input and another subset of the output that is unconstrained. In another aerosol application, Sturm et al.^11^ use scaling factors to ensure that a set of advected superspecies conserves total concentration in both particle and gas phases. However, none of these methods can be used to conserve atoms in data-driven models of atmospheric chemistry, which has additional complexity arising from molecular composition. For example, a scaling factor for each element cannot be used to conserve atoms, as molecular species are formed by mixtures of atoms from these different elements.

The mass-conserving framework for atmospheric chemistry introduced in Sturm and Wexler,^13^ based on relating atom fluxes to chemical tendencies, can be used to enforce atom conservation as a hard constraint for machine learning models. This framework serves as a machine-learning analog to traditional finite volume numerical methods.^13−16^ However, for atom conservation this framework requires either specialized data on chemical fluxes or custom graph neural network architectures^17−21^ embedded with a bipartite network relating reactions to species.^22^ Recent work by Döppel and Votsmeier^23^ point out that the detailed stoichiometry and reaction network connectivity is not known in some contexts. Requirement of specialized training data on fluxes, machine learning architectures, or knowledge of the reaction network could limit the more widespread adoption of this mass-conserving framework. For example, this framework is not directly compatible with physics-based models or other machine learning methods for predicting atmospheric composition like random forests^24^ or XGBoost^25^ emulating the GEOS-Chem mechanism,^26^ where every species has its own dedicated machine learning model trained to predict concentration, bias, or tendency. Such models require a different strategy for enforcing conservation.

This work introduces a corrective approach that minimally adjusts the predicted concentrations of chemical species to satisfy a set of elemental conservation laws exactly. This model-agnostic approach uses a single matrix multiplication step to nudge predictions to the closest physically consistent solution. We demonstrate this corrective approach on the gradient-boosting^27^ machine learning algorithm XGBoost^28^ that is trained to emulate a reference model of ozone formation.^13^ We derive a weighted version of this nudging approach informed by species-level uncertainty, preferentially adjusting species with lower accuracy and larger changes to meet the strict elemental conservation laws.

## 2 Methods and Model Description

### 2.1 Nudging Tendencies for Conservation during Incremental Change

The following section lays out a mathematical framework for ensuring any numerical prediction conserves atoms. In a chemical system with *m* compounds formed by *p* elements, a vector of species tendencies Δ*C* represents the concentration change of each species over a given time step Δ*t*: this real-valued vector  of *m* elements can be denoted as . The atomic composition of each species is defined by composition matrix *M*, which is an *m* by *p* matrix of positive integers , denoted as . If atom conservation is met by a particular predictive model, then conservation of atoms can be stated mathematically as
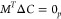
1where 0_*p*_ is a *p*-element zero vector. If instead there is an unphysical prediction Δ*C*′ where eq 1 does not hold, this means that the total number of atoms for one or more elements is changed by this prediction: the prediction artificially removed mass from or introduced mass to the system in an unphysical way.

We seek a way to minimally nudge Δ*C*′ to Δ*C* such that atoms are conserved. Mathematically, this means the constraint  in eq 1 is satisfied. We can recognize this as a constrained optimization problem, where we want to minimize the difference between nudged, mass-conserving solution Δ*C* and the original prediction Δ*C*′. If we define *minimal* to be with respect to the squared difference between these two predictions, this optimization represented mathematically is . With the choice of the L2 norm and equality constraints, finding the optimal nudge becomes a constrained least-squares problem with a closed-form solution.^29^ The minimally nudged mass-conserving Δ*C* to any unphysical Δ*C*′ can be obtained by a single matrix operation:
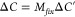
2where the correction matrix  that projects any Δ*C*′ onto the closest mass-conserving Δ*C* is defined by
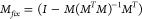
3

The derivation for this closed-form solution using the method of Lagrange multipliers is shown in a Supporting Information section S1 as a special case of the more general weighted least-squares optimization problem (section 2.2). Note that the constraints on positivity are removed for *M*_*fix*_, due to the subtraction from I and the use of the Moore-Penrose pseudoinverse . The Moore-Penrose pseudoinverse itself can give positive or negative values: negative values are unphysical for some applications such as extents of forward reaction^13^ or superspecies concentrations.^11^ Negative tendencies that satisfy  elementwise are still valid (though tendencies without flux constraints can sometimes lead to negative concentrations^21^). This method could also be modified to nudge absolute concentrations rather than incremental changes (tendencies): this approach is detailed in the Supporting Information section S2.

We note that the composition matrix *M* is used to ensure atom conservation in another related machine learning application, where the null space of *M*^*T*^ is used as a balance layer in an atom-conserving chemical reaction neural network used to discover stoichiometries in reaction systems.^23^ Analogously, the corrective approach in eq 3 nudges Δ*C*′ to be in the null space of *M*^*T*^, acting on concentrations instead of stoichiometric weights. The approach as tested here is a corrective nudge on predictions, rather than a built-in deep learning constraint, though it could also be built into neural networks for atom conservation in future applications.

Though this constrained least-squares nudging approach is straightforward and computationally feasible in its closed form, it is sparsely used in related literature in the atmospheric and chemical sciences. Least squares methods are sometimes used in solving the Stokes equations for fluid flow problems, but some of these approaches only conserve mass in the “weak sense” as part of the minimization problem rather than the hard constraint.^30,31^ Such soft constraint approaches are analogous to the more recent physics-informed neural networks,^32^ which encourage adherence to conservation laws or other known physics^8,33^ as regularization terms in the training loss function, often minimized with respect to squared error. Projection-based approaches for hard constraints in neural networks have been developed for more general applications outside of the atmospheric sciences.^34,35^ In the atmospheric sciences, Brown^36^ developed a least-squares nudging approach for mass conservation as a hard constraint in collisional breakup of raindrops, for which mechanisms and appropriately mass-conserving numerical methods are still being developed.^37^ Brown^36^ found that this least-squares nudging approach improved model comparisons to droplet spectra compared to using scaling factors for mass conservation. While we develop this nudging approach for atom conservation in atmospheric chemistry models, we note that this is readily generalizable to other applications in the intersection of physical sciences and data-driven numerical methods.

### 2.2 Species-Level Weights to Factor in Scale and Uncertainty

In the atmospheric chemical system, species span a wide range of concentrations, lifetimes, uncertainty, and societal relevance. We adjust the method in section 2.1 to assign *species-level* weights in the optimization equation, resulting in the weighted least-squares optimization problem (with the hard constraint *M*^*T*^Δ*C* = 0_*p*_ unchanged)
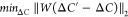
4where  scales the adjustment of each species by some measure of its importance. The higher the species-level weight, the less it is adjusted in the correction to atom conservation. With this adjustment, the weighted correction matrix becomes
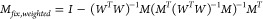
5

In the case that *W* is identity, or a diagonal matrix with equal weights, the problem simplifies to the unweighted case in section 2.1. The derivation of the weighted correction approach using Lagrange multipliers, and the special case of the unweighted approach, is shown in section S1 in the Supporting Information.

The choice of the species-level weighting is flexible as long as the weights remain positive, and could be hand selected using domain knowledge, scaled by the relative tendencies of the species, or determined from a measure of uncertainty of the species prediction. As a proof of concept, we consider both the magnitude of tendencies and the uncertainty in prediction with the species-level weight:
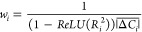
6where  is just a scaling factor equal to the average absolute tendency of species *i*, to account for the wide spread in magnitude of tendencies. Species-level uncertainty, defined in this context as error of the ML predictions with respect to the ground truth (the reference model), is represented by the  factor.  is the coefficient of determination between the predicted tendencies and the reference model. *ReLU(x)* is the rectified linear unit function, defined as  to ensure the positivity constraint on *w*_*i*_ if species *i* is very poorly predicted. The species-level weights *w*_*i*_ compose the values of the diagonal matrix *W*.

On a scaled basis using , we note that weighted approach converges to an atom-conserving extension of the completion method in Harder et al.,^12^ in the event that one variable is by far the worst performing or if the species-level weight is made to be more sensitive to uncertainty than in eq 6.

An advantage of the closed-form approach in eqs 3 and 5 is that the correction matrix need only be calculated once. Then, a single matrix operation can be used to correct all future predictions. This computational cost is comparable to a simple multilinear regression model predicting all species tendencies, or a single hidden layer in a neural network predicting all species tendencies. Tree-based methods like XGBoost are based on depth and number of ensemble members but would likely be more computationally costly than a single matrix operation. For other numerical approaches, this correction requires as many floating point operations as a forward pass of an exact Jacobian or its inverse, and could require fewer operations than a single internal step of a Rosenbrock solver, which requires inversion of an equally sized Jacobian matrix (though this often leverages sparse inversion methods).^4^ The computational tractability of the mass-conserving nudge operation, after one-time construction of the correction matrix, could allow this approach to scale with more complex systems.

### 2.3 Example Using the Primary Photolytic Cycle

We use a fundamental cycle in atmospheric chemistry to demonstrate the projection approach. The primary photolytic cycle between nitrogen oxides and ozone in the troposphere, from which the Leighton relationship can be derived, is shown in Table 1.

**Table 1:** Primary Photolytic Cycle

Reaction	Reaction Number
NO_2_ + hv → NO + O	R1
O + O_2_ → O_3_	R2
O_3_ + NO → NO_2_ + O_2_	R3

The matrix  is a composition matrix of *m* compounds formed by *p* elements, *m* = 5 and *p* = 2 in this example, and
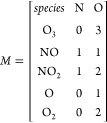


**Figure 1:**
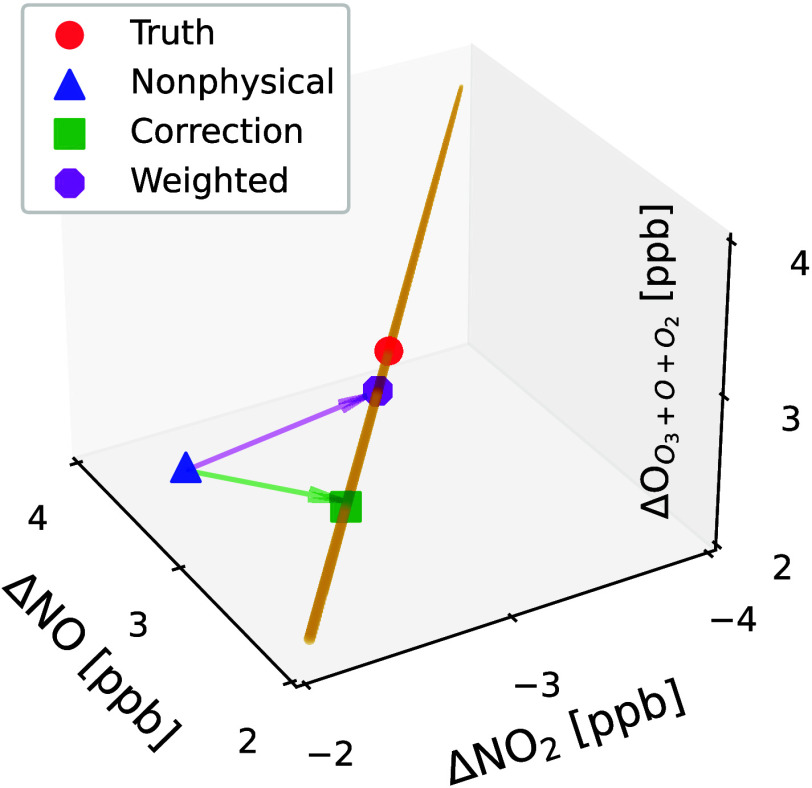
Visualization of the corrective approach for the primary photolytic cycle. The 3 physically consistent predictions (truth, correction, and weighted nudge) lie on a yellow line that represents the conservative manifold on which both nitrogen and oxygen atoms are conserved.

We demonstrate the following example of both nudging approaches, visualized in Figure 1. In this example,  is a vector of tendencies for the 5 different molecular species: if R1 proceeds at 5 ppb/min, R2 at 4 ppb/min, and R3 at 2 ppb/min, then after 1 min (assuming rates do not change) the tendencies are  in ppb, as shown by the red circle in Figure 1. If instead an incorrect unphysical vector  is predicted as shown by the blue triangle, the prediction is moved off of the physically possible constrained manifold (the yellow line) and  meaning there are 1 ppb nitrogen atoms and 1.62 ppb oxygen atoms artificially added to the system. In this example, , correcting the mass imbalance to the green square in Figure 1.

Let us now factor in uncertainty and assume that there is a spread of accuracy across variables on a test data set, where we find NO is much better predicted than NO_2_, and O_3_ has better predictions than atomic or diatomic oxygen. We apply the uncertainty-weighted correction from eq 5 to nudge the prediction to a more likely mass-conserving solution. The accuracies of this fictional test data set yield R^2^ values of 0.99 for O_3_, 0.97 for NO, 0.92 for NO_2_, 0.92 for O, and 0.92 for O_2_, where we assume equal scaling factors in this example. In this case,  correcting the blue triangle in Figure 1 to the purple octagon, which is now mass balancing (lying on the physically possible manifold). While not the closest mass-conserving solution to Δ*C*′, this weighted correction is much closer to the original solution because it nudged the species that were most uncertain, while leaving likely accurate species like NO and O_3_ almost untouched.

### 2.4 The Julia Photochemical Mechanism

To evaluate this method in a real use case, we use the Julia photochemical model^13^ as a reference model to train a machine learning XGBoost emulator. The Julia photochemical model is a simplified mechanism containing the reactions and species necessary for troposphere ozone photochemistry including NO_*x*_ cycling, radical chemistry, and VOC oxidation. It has been used as a reference model for several data-driven and machine learning algorithms, including neural networks,^21^ sparse identification of nonlinear dynamics^38,39^ and data-driven discovery of conservation laws.^40^

**Figure 2:**
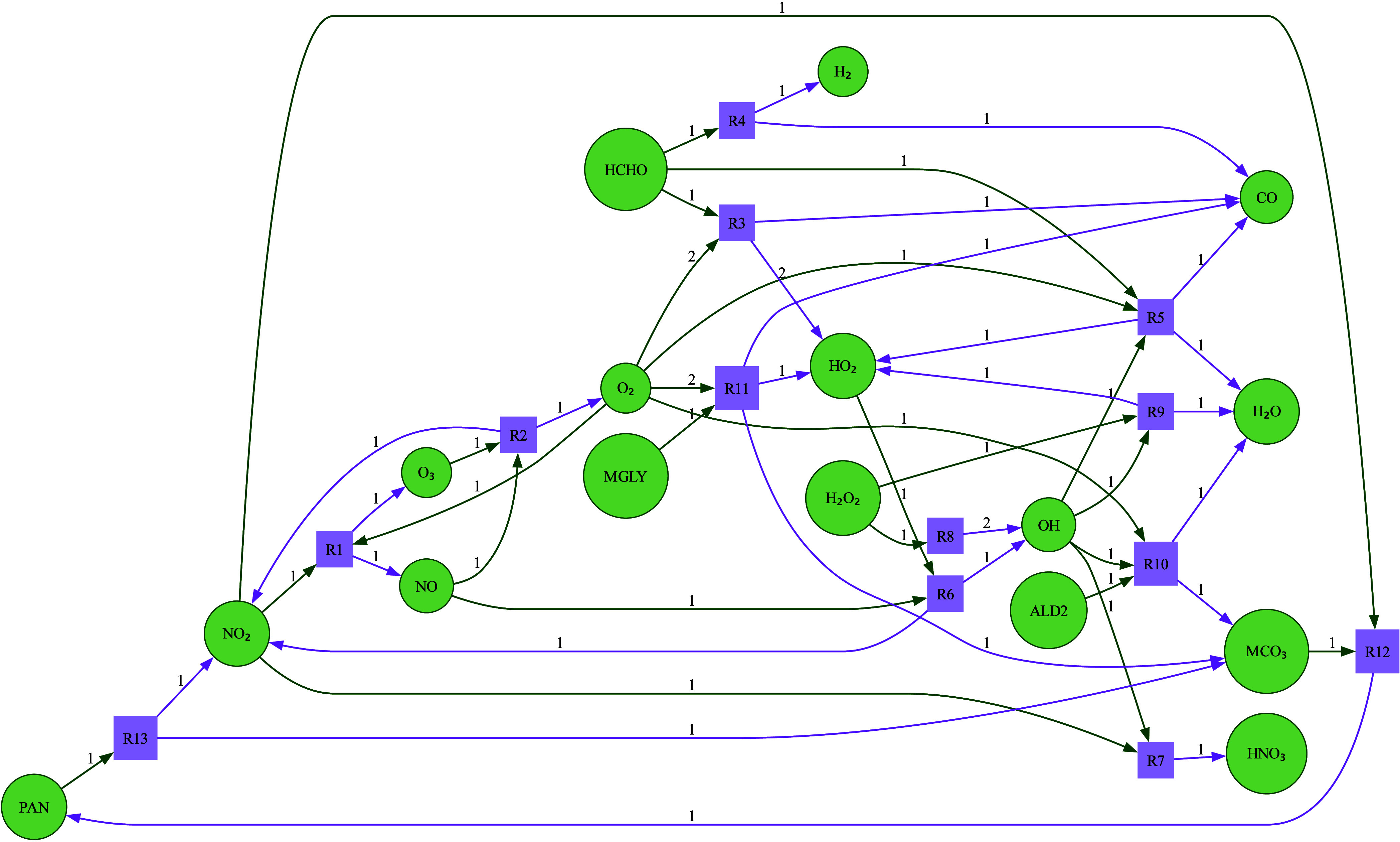
Updates to the Julia photochemical mechanism include peroxyactyl nitrate (PAN) chemistry, as well as important precursors and associated radicals. The numbers on the arrows denote stoichiometric weights. The full reaction list is contained in S3 in the supplement.

Prior hard constraints work focused on carbon and nitrogen conservation during ML emulation of an older version of the Julia photochemical model which, without a carbon and nitrogen mixed-element species and ignoring hydrogen and oxygen balances, could be solved with two independent scaling factors. To demonstrate how this corrective approach automatically handles a mixed-element carbon and nitrogen species, for the rest of this work we use a version of the model augmented to include peroxyacetyl nitrate (PAN), which has the chemical formula CH_3_COO_2_NO_2_. We update the Julia photochemical mechanism to include PAN and associated reactions, visualized in the mechanism graph in Figure 2. We include two important precursors of PAN, acetaldehyde and methylglyoxal,^41^ as well as the intermediate peroxyacetyl radical CH_3_C(O)OO. We denote acetaldehyde, methylglyoxal, and peroxyacetyl radical as ALD2, MGLY, and MCO_3_ respectively in our mechanism for consistency with the GEOS-Chem atmospheric chemistry module.

We include updates from Yang et al.^39^ who further developed the Julia photochemical model to conserve all atoms, not just carbon and nitrogen, and construct it using the Julia package Catalyst.jl.^42^ With this package, the time evolution of the chemical system can be simulated using the Julia library DifferentialEquations.jl,^43^ which includes a suite of stiffly stable solvers. The reference model is inherently conservative, both in its stoichiometry as well as use of the exact Jacobian in the Rosenbrock solver for integration,^4^ with deviations of all training and test data within <10^–8^ ppb for oxygen atoms and <10^–12^ ppb for atoms of all other elements. Besides the PAN-related expansion, another change between the new version of the model in the present work and the updates in Yang et al.^39^ is that atomic oxygen is removed, as well as its reaction with diatomic oxygen. This is a minor structural difference to the original mechanism in Sturm and Wexler,^13^ which by applying a pseudosteady state assumption to atomic oxygen to reduce numerical stiffness, ensured that the atomic oxygen reaction proceeded at the same rate as NO_2_ photolysis, effectively lumping the first two reactions together. We now represent these two reactions as a single reaction. We also ensure that none of the reaction rates include diatomic oxygen concentration as a factor in their rate laws, instead using the effective rate constant for termolecular reactions from the JPL 19–5 data evaluation “Chemical Kinetics and Photochemical Data for Use in Atmospheric Studies”.^44^ We update all bimolecular and termolecular reactions to use rate constants recommended by JPL 19–5.^44^ All photolytic rates are obtained using the KPP Standalone Interface within a GEOS-CF run,^45^ at a surface grid cell containing Los Angeles used in prior work exploring chemical cycling in the atmosphere.^46^

We use the Julia photochemical model in the above configuration to simulate 1.1 million 1-h runs, with tendencies reported every 5 min. The application focused on in this paper is in ML emulation of the short time scales of this photochemical system: for a larger system in a longer simulation, which can have day-to-month time scales important to other aspects of atmospheric chemistry, this approach would have to be evaluated and perhaps adjusted with respect to prediction targets or weighting strategy. As in prior work using this reference model, concentrations are randomly initialized within realistic ranges for every case.^21,47^ We train the XGBoost algorithm to predict 5 min tendencies of all species as targets, given species concentrations as input. In this multitarget regression problem, the tendency of each species is given its own XGBoost predictor, each of which is composed of 1000 estimators subsequently boosting the gradient with respect to mean squared error and a learning rate of 0.01 scaling the predictions of each estimator. Each estimator itself is a decision tree structure with a maximum depth of 10 levels. All other hyperparameters besides a random seed keyword argument are kept at package defaults (documentation available at https://xgboost.readthedocs.io/en/stable/, last access August 27, 2024). We use 90% of the cases for training and reserve 10% (110,000 1-h runs) for testing in the following results section.

## 3 Results

### 3.1 Mass Imbalance of XGBoost Predictions

**Figure 3:**
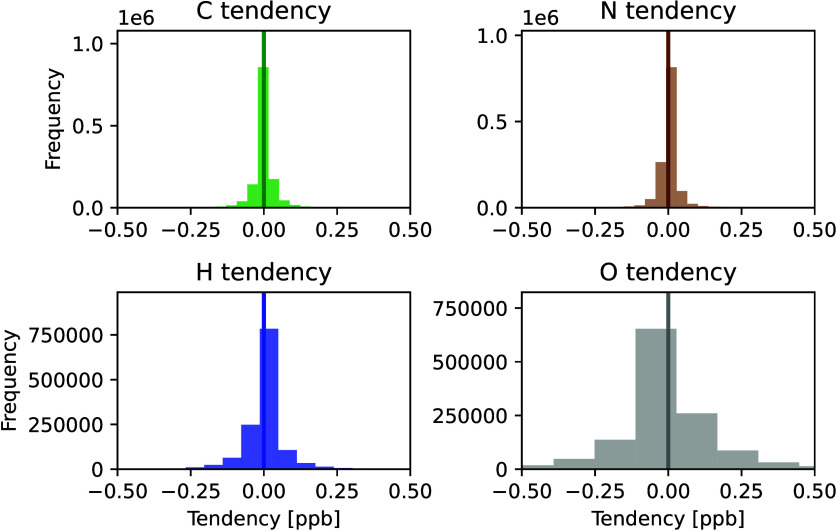
Distributions of the atom imbalance for each element.

Before applying the corrective nudging approach, we first assess the extent to which atoms are not conserved in the XGBoost predictions. Figure 3 shows the distributions of the mass imbalances, based on the net tendencies (change in concentration over 5 min) of each element. The tendency using the corrective method is plotted as a vertical line at 0, as after nudging net atom tendencies are all within 10^–13^ ppb, more conservative than the original reference model (for further analysis of the precision extent of conservation see section S4 and Figure S1).

For all elements, the distributions are near zero-centered, which indicates low bias (atoms are not, on average, added to or removed from the system in large amounts) and is consistent with the good performance of XGBoost for prediction in the atmospheric chemical sciences.^25,48−50^ As a rough visual estimate, the majority of all the distributions remain within smaller bounds on the order of hundreds of parts per trillion (tenths of ppb), which is ca. 10% the size of 5 min tendencies for many of the species. This underscores that these unphysical deviations from machine learning models can often be minor, in which case the predictions only need a small nudge to a solution that conserves mass.

More quantitatively, the XGBoost 5 min predictions have maximum mass imbalances of 2.2 ppb for carbon, 2.1 ppb for nitrogen, 3.4 ppb for hydrogen, and 7.1 ppb for oxygen. The average atom imbalances from XGBoost are smaller, at 0.023 ppb for carbon, 0.025 ppb for nitrogen, 0.045 ppb for hydrogen, and 0.156 ppb for oxygen atoms, many orders of magnitude larger than the maximum imbalances of the reference model. These average imbalances are on the order of 1–10% of the 5 min tendencies for many species, with the exception of H_2_O_2_ and OH: the average imbalances are on the order of the average absolute tendency of H_2_O_2_, and 2 orders of magnitude larger than the average absolute tendency of OH. This motivates a scaled approach in the correction, to not balance atoms at the cost of accuracy for species with small tendencies.

### 3.2 Effect of Nudging on Accuracy

In line with the relatively minor unphysical atom conservation deviations shown in section 3.1, the XGBoost ensemble is able to predict the tendencies of all individual species well, with high R^2^ and low error for all species (shown in section S5 in the supplement, Figures S2–S4). The least accurately predicted species is OH, where performance statistics are still reasonably good on test data (R^2^=0.9428, RMSE = 4 × 10^–4^ ppb). This result allows us to ask a targeted question: does the corrective nudging approach decrease the quality of predictions, or phrased differently, does this approach ensure mass conservation at the cost of accuracy? The following results indicate that the unweighted nudge does indeed sacrifice accuracy for physical consistency, while the weighted nudge actually improves accuracy slightly while transforming the predictions to conserve mass.

**Figure 4:**
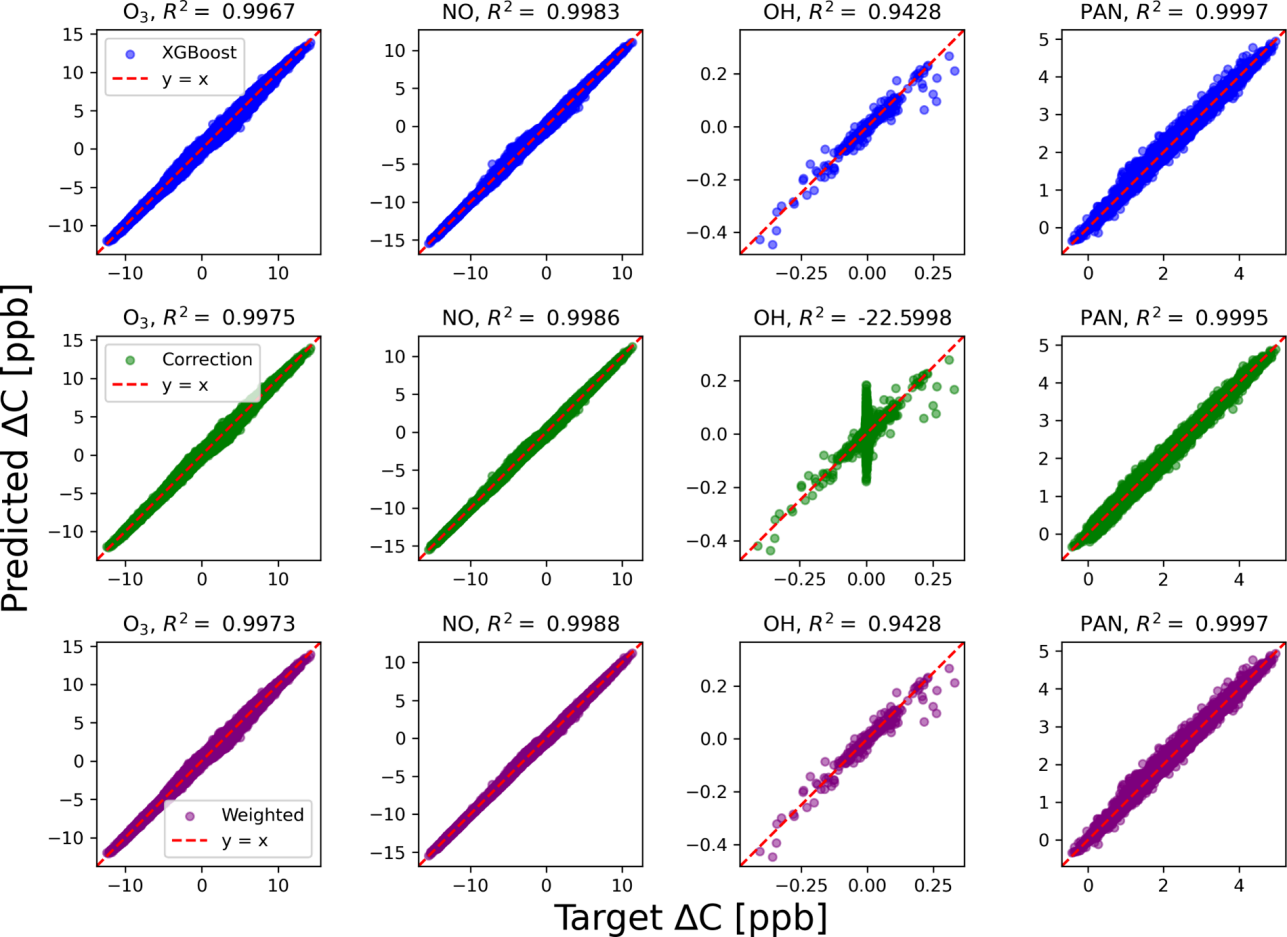
Scatter plot of key species with the uncorrected predictions, corrected predictions and the weighted correction. The negative R^2^ value for OH in the unweighted correction (middle row) arises from the error residuals being larger than the total sum of squares, meaning that after the unweighted corrections, predictions of OH are less accurate than simply predicting average OH tendency every time.

Figure 4 shows the accuracy of predictions for four representative species: O_3_, NO, OH, and PAN. The uncorrected prediction accuracy with respect to the reference model is shown on the first row, the nudging approach on the second row, and the species-level weighted approach on the third row. The carbon and nitrogen mixed species PAN is already very well predicted before applying constraints and only receives a slight accuracy loss with the unweighted correction in row two, largely stemming from perturbations to smaller tendencies. The very accurate predictions of O_3_ and NO are barely changed by the adjustment in the second row, though slightly improved.

The nudging correction in the second row obliterates the accuracy of OH predictions, which have much smaller absolute tendencies. The difficulty of capturing OH was also seen in prior work using hard-constrained deep learning embedded with the mass-conserving framework.^21^ As in previous work, we could design the approach for this specific system to neglect conservation of oxygen and hydrogen, which have diatomic oxygen and water as extremely large source/sink terms; in that case, we could just remove columns of *M* and leave OH unperturbed. However, we seek a more general framework that can handle radicals, as some applications may have radicals or other important species that are mixtures of multiple elements that need to be conserved: for example, reservoir chlorine nitrate is an important sink and source of reactive chlorine and nitrate which are in turn sinks of stratospheric ozone.^51−53^ The performance of OH in the second row indicates that the unweighted nudging approach may not be appropriate for predictions that span orders of magnitude across different variables. Though O_2_ makes the largest perturbation to OH in this approach, all other species make disproportionate adjustments to it as well. On average, other species perturb OH anywhere from 10 times to 630 times the magnitude of its average tendency. We turn to the species-level weighting approach to address this scaling issue.

We design the species-level weighting approach and corresponding weighting function to be able to handle predictions of small radicals. The third row of Figure 4 shows that OH accuracy is not adversely affected when using the species-weighted nudging approach. That is because the species-level weighting approach contains two factors, a scaling factor and uncertainty factor. The first factor is simply a scaling factor to account for the magnitude-spanning spread of tendencies across different species. This scaling factor in the species-level weighting allows for small radicals to be well predicted after the nudge, in this case OH. In other words, the species-weighted nudge prevents adjustment of OH far from its predicted tendency (relative to its average tendency), because it varies by smaller amounts than other species.

The second factor represents the uncertainty in species predictions as inversely related to R^2^ of the uncorrected approach on a test data set. This uncertainty factor, with weights appropriately chosen, encourages the global solution to be more accurate. The overall R^2^ of all species besides OH is 0.9987 for the uncorrected predictions and the unweighted approach degrades predictions during the nudge to an R^2^ of 0.9929 for all species besides OH. In contrast, the weighted approach improves predictions slightly during the nudge to an R^2^ of 0.9989 for all species besides OH. This is expected behavior and happens by design, by definition of uncertainty as likelihood of incorrect prediction by the ML method. Rather than nudging to whatever is the closest prediction (from a least-squares perspective) that conserves mass, this approach considers the uncertainties in the ML predictions of different species to nudge to a more likely mass-conserving solution.

### 3.3 Further Exploration of Species-Level Weighting

**Figure 5:**
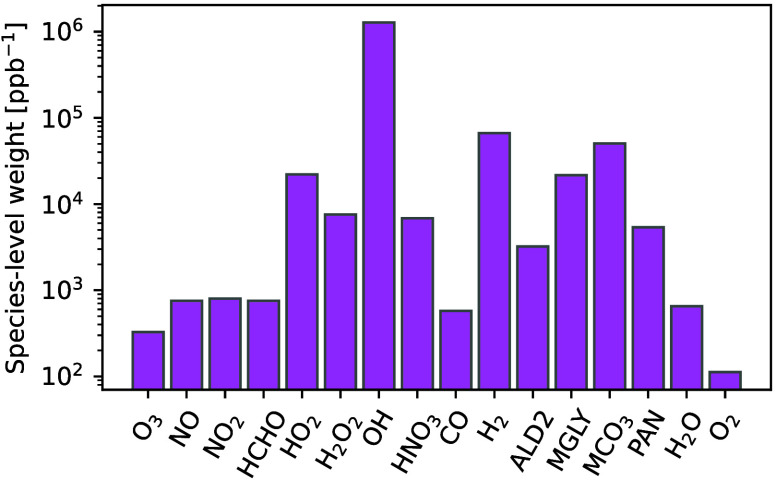
Species-level weights used in the weighted correction.

The species-level weights improve both OH radical prediction and the global metrics of the solution. Figure 5 visualizes these weights, which are comprised of both a scaling factor and an uncertainty factor. We note that OH is the most heavily weighted species, followed by H_2_, reflecting a balance between the uncertainty and scaling factor. The 3 next most heavily weighted species are MGLY and the radicals MCO_3_ and HO_2_. The values of these weights are not universal, as they are determined by the behavior of the system as represented by the training data. This requires the training data to be representative for both factors in eq 6. The training data does have representative average tendencies, but the R^2^ for OH is much higher than in the test data set at 0.9971 (though among the lowest for training R^2^ of all species, approximately tied with that for O_3_), indicating that the XGBoost model is somewhat overfit with respect to OH. Other approaches to species-level weighting are possible, including composition- and tendency-dependent weighting or manual selection of importance. Improved global accuracy and preservation of OH predictions shows that eq 6 is appropriate for the photochemical system investigated here, automatically weighing both scale and uncertainty in XGBoost predictions.

**Figure 6:**
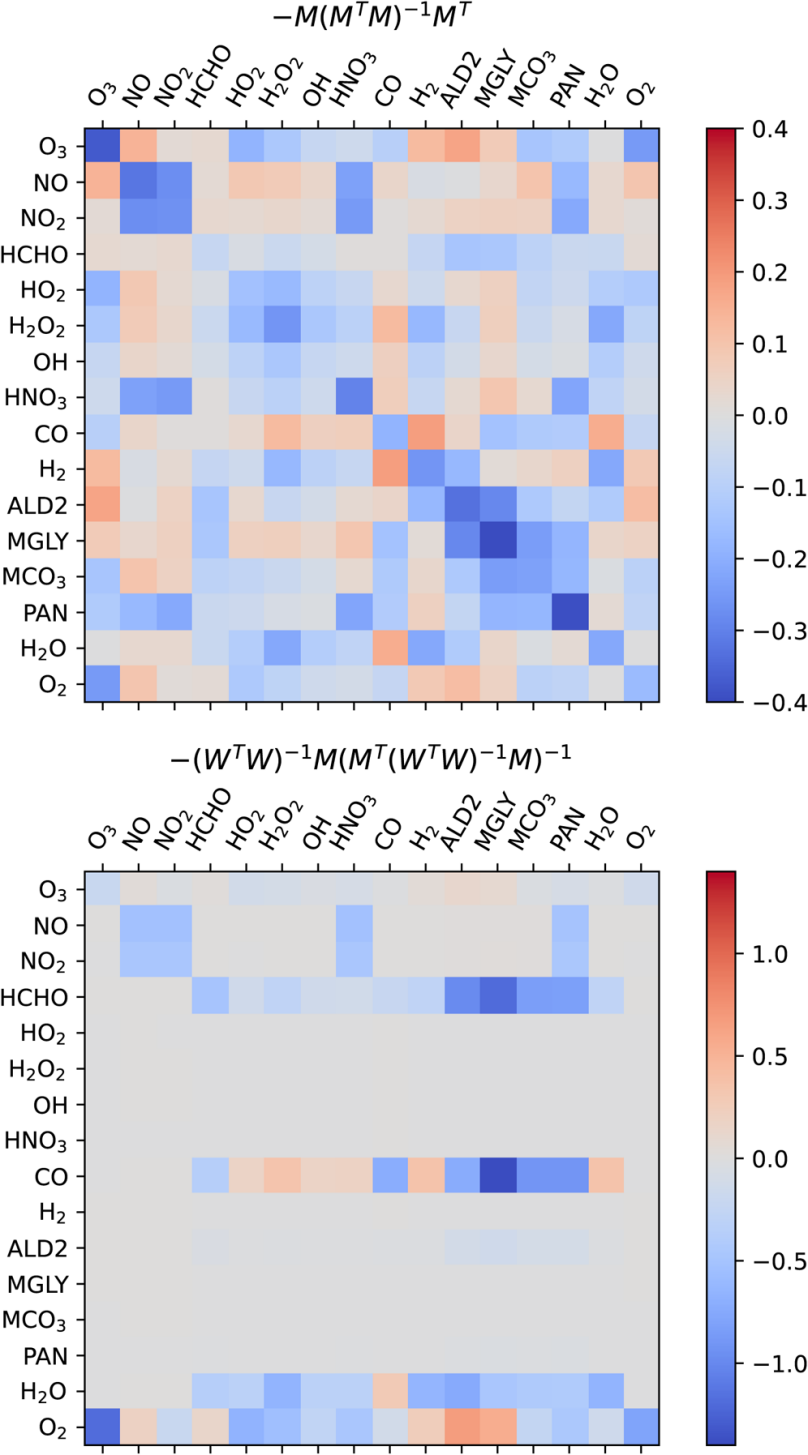
Term of the projection matrices determining the perturbation to Δ*C*′. Note that these are added to the identity terms to create the nudging projection matrices *M*_*fix*_ and *M*_*fix,weighted*_.

Figure 6 visualizes the parts of the projection matrices that determine the perturbation to Δ*C*′. This is the nonidentity part of the correction matrix in eqs 3 and 5 for the unweighted and the weighted approaches respectively (for visual purposes, the identity component adding 1 to the diagonals is omitted). The unweighted matrix is not data-driven, but rather a static property emerging from the molecular formulas in the system. The weighted matrix is data-driven in that it depends on the training data uncertainty in predictions and average absolute tendencies.

The unweighted projection is fully symmetric, as is the perturbation component of it (within a relative tolerance of 10^–13^). The weighted approach is asymmetric and sparse. Species with high weights have essentially no perturbation, for example the OH row for the weighted matrix. This reflects that the choice of species-level weighting is automatically more targeted in the species that it nudges.

## 4 Conclusions

If not anchored by hard constraints, numerical predictions of atmospheric chemical behavior will yield unphysical results. This includes not conserving mass, or not conserving atoms in a machine learned chemical reaction network; models based on data rather than fundamental process knowledge are particularly prone to such unphysical results. These unphysical violations can be minor, requiring only a small adjustment. Our corrective approach based on least-squares finds the smallest possible nudge to the predictions that guarantees conservation of atoms to machine precision.

When tested on an XGBoost-predicted ozone photochemical system, where the changes of different species span orders of magnitude, we find that species-level weights are essential for an accurate correction. A scaling factor needs to be introduced to accurately model hydroxyl radical, an important species with low absolute concentrations. An uncertainty factor in the species-level weights improves the overall accuracy in predictions. Rather than making the smallest possible adjustment to the predictions, the weighted approach uses species-level uncertainty (defined here as error in ML predictions on the training data set) to target which species to adjust. For the photochemical system studied here, species-level weights in the correction lead to a moderate improvement in overall accuracy. The weighted correction guarantees physical consistency at no cost to accuracy, instead slightly improving accuracy of most species in the mechanism. We expect this method to generalize to other systems, including larger mechanisms with longer time scales, though some adjustments may be necessary with respect to prediction targets or weighting strategy.

The atom-conserving nudge can act as a wrapper for any numerical approach and does not require custom architectures^21^ or training data^13^ as in prior efforts. Included in this wrapper can be a calculation of tendencies before subsequent correction if the predictions are concentrations. Only the elements of interest need to be constrained such that their atoms are conserved, shown in the comparison of corrective vs flux-based approaches in S6 in the Supporting Information. For this reason, the approach is well-suited as an automated unit test to test mass closure in larger chemical mechanisms and could also be paired with an automatic fix if the numerical solvers do violate conservation of atoms for key elements. A future extension of this method could augment the composition matrix to include species in different grid cells, for example to correct mass conservation issues stemming from transport in 3D models. Beyond numerical models, this wrapper has potential application to experimental chamber chemistry data analogously to the approach in Brown^36^ that improved model-measurement agreement when applying a least-squares mass correction in a raindrop collision application. For example, empirical data often have closure issues from processes like wall losses^54^ and protocols have been developed to standardize chamber wall loss characterization experiments.^55^

This uncertainty-weighted nudging approach is not limited to being used as a corrective wrapper: it could be built into a neural network layer as another option for automatic hard constraints in deep learning, alongside prior approaches in the Earth system sciences^8−10,21^ and more generally in the physical sciences,^14,18,19,23,56−58^ as well as an alternative way of representing and handling uncertainty in deep learning.^14,59−61^ Potential architectures this atom-conserving nudging approach could be built into are deep learning autoencoders integrating a chemical mechanism forward as in Kelp et al.^47,62^ but bounded in a stoichiometrically resolved and mass-conserving latent space, or neural networks mapping between low and high chemical complexity while conserving the total budget of atoms.

Beyond atom conservation, the uncertainty-weighted approach could be used in other applications combining variable-level uncertainty with hard constraints to nudge predictions to be both physically consistent and more accurate.

## Data Availability

The exact versions of the scripts and model output used for analysis and figures in this work are available at 10.5281/zenodo.13385987; the exact version of the Julia photochemical model used to produce the model output, as well as the modifications to Catalyst.jl for Figure 2, is available at 10.5281/zenodo.14027350.

## Supporting Information Available

S1, Derivation of the approach using Lagrange multipliers; S2, Modification for concentrations instead of tendencies; S3, Julia photochemical model reaction list; S4, Precision extent of conservation; S5, Scatter plots of accuracy for all other species; and S6, Comparison to another hard constraints framework (PDF)

## Abbreviations

ALD2: acetaldehyde
GEOS: Goddard Earth Observing System
GEOS-CF: Goddard Earth Observing System Composition Forecasts
MCO_3_: peroxyacetyl radical
MGLY: methylglyoxal
PAN: peroxy acetyl nitrate
ReLU: rectified linear unit
RMSE: root-mean-square error
XGBoost: extreme gradient boosting
