# Supplementary material for: A Nudge to the Truth: Atom Conservation as a Hard Constraint in Models of Atmospheric Composition Using a Species-Weighted Correction
Source: ACS EST Air. 2024 Nov 20;2(1):99–108. doi: 10.1021/acsestair.4c00220 (PMC11730974; doi:10.1021/acsestair.4c00220)
Supplement: Supplementary file 1 — ea4c00220_si_001.pdf [file ea4c00220_si_001.pdf]

Supporting Information for

A nudge to the truth: atom conservation as a hard constraint in models of atmospheric composition using a species-weighted correction

Patrick Obin Sturm (psturm@usc.edu) and Sam J. Silva

S1. Derivation of the weighted correction approach using Lagrange multipliers

We want to find the best nudge to a set of concentration changes of chemical species such that atoms in the system are conserved exactly.

Mathematically, we seek an optimal correction $\Delta C \in \mathbb{R}^m$ to a set of m species concentrations $\Delta C' \in \mathbb{R}^m$, where the species are formed by atomic combinations of $p \leq m$ elements in $M \in \mathbb{Z}_{\geq 0}^{m,p}$ such that no atoms from any of the elements are added or removed in a nonphysical way. To keep it general, we assign species-level weights $W \in \mathbb{R}_{\geq 0}^{m,m}$ which determine the importance of each species; important species are adjusted less. In the case that W is the same for all species, then this is an unweighted approach that finds the smallest correction.

This can be framed as a constrained optimization problem:

$$min_{\Delta C} \|W(\Delta C' - \Delta C)\|_2 \qquad \text{S1.1}$$

Subject to the constraints

$$M^T \Delta C = 0_p \qquad \text{S1.2}$$

We note that other constraints could be added, e.g. element-wise inequality constraints, $-M_{fix}\Delta C' \leq C$ for all $\Delta C' < 0_m$, but neglect these in order to be able to obtain a closed-form solution. We also note that other measures of distance besides the L2-norm exist but use this norm for the same purpose of obtaining a closed-form solution. We use the method of Lagrange multipliers to obtain the solution to this constrained least squares problem[1], beginning with setting up the Lagrangian function:

$$\mathcal{L}(\Delta C, \lambda) = (\Delta C' - \Delta C)^T W^T W(\Delta C' - \Delta C) + \lambda^T(M^T \Delta C) \qquad \text{S1.3}$$

Where the Lagrangian $\mathcal{L}$ is a scalar function that combines the original objective function (the first term is S1.1 squared) with the constraints as a second term, weighted by the Lagrangian multiplier $\lambda \in \mathbb{R}^p$.

We seek the ΔC where the derivative of the Lagrangian function with respect to ΔC is zero:

$$\frac{\partial \mathcal{L}}{\partial \Delta C} = 2W^T W(\Delta C' - \Delta C) + M\lambda = 0_m \qquad \text{S1.4}$$

that simultaneously obeys the constraint in S1.2, which holds where the derivative of the Lagrangian function with respect to λ is zero:

$$\frac{\partial \mathcal{L}}{\partial \lambda} = M^T \Delta C = 0_p \qquad \text{S1.5}$$

We now use these two equations to solve for the optimal, atom-conserving ΔC. We rearrange S1.4 to isolate $\Delta C' - \Delta C$:

$$-\frac{1}{2}(W^TW)^{-1}M\lambda = \Delta C' - \Delta C \qquad \text{S1.6}$$

Then, we multiply both sides by M^T, keeping in mind that $M^T\Delta C = 0_p$:

$$-\frac{1}{2}M^T(W^TW)^{-1}M\lambda = M^T\Delta C' - \cancel{M^T\Delta C} \qquad \text{S1.7}$$

Allowing us to isolate λ:

$$\lambda = -2(M^T(W^TW)^{-1}M)^{-1}M^T\Delta C' \qquad \text{S1.8}$$

We can substitute λ for equation S1.8 in equation S1.4:

$$\frac{\partial \mathcal{L}}{\partial \Delta C} = 2W^TW(\Delta C' - \Delta C) - 2M(M^T(W^TW)^{-1}M)^{-1}M^T\Delta C' = 0_m \qquad \text{S1.9}$$

Dividing by 2 and isolating the term with ΔC, we get

$$W^TW\Delta C = W^TW\Delta C' - M(M^T(W^TW)^{-1}M)^{-1}M^T\Delta C' \qquad \text{S1.10}$$

To isolate the optimal nudge ΔC, we multiply both sides by $(W^TW)^{-1}$:

$$\Delta C = \Delta C' - (W^TW)^{-1}M(M^T(W^TW)^{-1}M)^{-1}M^T\Delta C' \qquad \text{S1.11}$$

And to get this in the form of a single matrix multiplication, we factor $\Delta C'$ out of the right-hand side to obtain

$$\Delta C = (I - (W^TW)^{-1}M(M^T(W^TW)^{-1}M)^{-1}M^T)\Delta C' \qquad \text{S1.12}$$

Where I is the identity matrix of size m and we define the weighted correction matrix as in section 2.2 in the main text:

$$M_{fix,weighted} = I - (W^TW)^{-1}M(M^T(W^TW)^{-1}M)^{-1}M^T \qquad \text{S1.13}$$

We note that if we multiply S1.11 by M^T, the right-hand side becomes $M^T\Delta C' - M^T\Delta C' = 0_p$, satisfying S1.2 to conserve atoms. We also note that if the weight matrix is equal to identity or a diagonal matrix with equal elements, or if it is orthonormal, the problem simplifies to the unweighted correction in section 2.1 of the main text. We finally note that there can be at maximum as many elements as species, $p \leq m$, or more general up to as many constraints as variables, in order to allow invertibility in the above approach.

S2. Nudging concentrations to conserve total amounts

This approach could be augmented to nudge total concentrations, if concentrations instead of tendencies are predicted. To find the smallest perturbed concentration mathematically, we seek

$$min_C \|C' - C\|_2 \quad \text{S2.1}$$

subject to

$$M^T C = A \quad \text{S2.2}$$

where $C' \in \mathbb{R}_{\geq 0}^{m,1}$ is a prediction of m species chemical concentrations, $C \in \mathbb{R}_{\geq 0}^{m,1}$ is the corrected prediction, and $A \in \mathbb{R}_{\geq 0}^{p,1}$ is a vector containing the total atom concentrations of $p < m$ elements. $M \in \mathbb{Z}_{\geq 0}^{m,p}$ is the same composition matrix as in Section S1 and Section 2.1 in the main text.

The optimal C (optimal with respect to S2.1) that satisfies S2.2 is

$$C = C' + M(M^T M)^{-1}(A - M^T C') \quad \text{S2.3}$$

There are different strengths and weaknesses of adjusting concentrations versus tendencies. The tendencies approach has the advantage of ΔC not necessarily needing to remain positive. In addition, incremental changes are less likely to span many orders of magnitude. However, the tendencies or prior concentrations to calculate may not always be known in every application: an example of this is mapping back and forth between latent dimensionality and full dimensionality[2–5] or conserving a set of properties when converting between lumped superspecies and species[5].

S3. Julia photochemical mechanism

Table S1. Species	
Name	Species ID
Ozone	O_3
Nitric oxide	NO
Nitrogen dioxide	NO_2
Formaldehyde	HCHO
Hydroperoxyl radical	$HO_2^{\cdot}$
Hydrogen peroxide	HO_2H
Hydroxyl radical	$OH^{\cdot}$
Nitric acid	HNO_3
Carbon monoxide	CO
Diatomic hydrogen	H_2
Acetaldehyde	ALD2
Methylglyoxal	MGLY
Peroxyacetyl radical	MCO_3
Peroxyacetyl nitrate	PAN
Water	H_2O
Diatomic oxygen	O_2

The set of species in Table S1 corresponds to the following composition matrix M:

$$M = \begin{bmatrix} species & \mathrm{C} & \mathrm{N} & \mathrm{H} & \mathrm{O} \\ \mathrm{O_3} & 0 & 0 & 0 & 3 \\ \mathrm{NO} & 0 & 1 & 0 & 1 \\ \mathrm{NO_2} & 0 & 1 & 0 & 2 \\ \mathrm{HCHO} & 1 & 0 & 2 & 1 \\ \mathrm{HO_2} & 0 & 0 & 1 & 2 \\ \mathrm{H_2O_2} & 0 & 0 & 2 & 2 \\ \mathrm{OH} & 0 & 0 & 1 & 1 \\ \mathrm{HNO_3} & 0 & 1 & 1 & 3 \\ \mathrm{CO} & 1 & 0 & 0 & 1 \\ \mathrm{H_2} & 0 & 0 & 2 & 0 \\ \mathrm{ALD2} & 2 & 0 & 4 & 1 \\ \mathrm{MGLY} & 3 & 0 & 4 & 2 \\ \mathrm{MCO_3} & 2 & 0 & 3 & 3 \\ \mathrm{PAN} & 2 & 1 & 3 & 5 \\ \mathrm{H_2O} & 0 & 0 & 2 & 1 \\ \mathrm{O_2} & 0 & 0 & 0 & 2 \end{bmatrix}$$

Table S2: Julia photochemical mechanism		
Reaction Number	Reaction	Rate constant reference
R1	$NO_2 + O_2 + hv \rightarrow NO + O_3$	See note 1
R2	$O_3 + NO \rightarrow NO_2 + O_2$	JPL 19-5[6] page 84 Table 1C
R3	$HCHO + 2O_2 + hv \rightarrow 2\ HO_2^{\cdot} + CO$	See note 1
R4	$HCHO + hv \rightarrow H2 + CO$	See note 1
R5	$HCHO + HO^{\cdot} \rightarrow HO_2^{\cdot} + CO + H_2O$	JPL 19-5[6] page 106 Table 1D
R6	$HO_2^{\cdot} + NO \rightarrow OH^{\cdot} + NO_2$	JPL 19-5[6] page 83 Table 1C
R7	$OH^{\cdot} + NO_2 \rightarrow HNO_3$	See note 2, JPL 19-5[6] page 434 Table 2-1
R8	$HO_2H + hv \rightarrow 2\ OH^{\cdot}$	See note 1
R9	$HO_2H + OH^{\cdot} \rightarrow H_2O + HO_2^{\cdot}$	T-independent recommendation, JPL 19-5[6] page 73
R10	$ALD2 + OH + O_2 \rightarrow MCO_3 + H_2O$	JPL 19-5[6] page 107 Table 1D
R11	$MGLY + 2O_2 \rightarrow MCO_3 + CO + HO_2^{\cdot}$	See note 1
R12	$MCO_3 + NO_2 \rightarrow PAN$	See note 2, JPL 19-5[6] page 435 Table 2-1
R13	$PAN \rightarrow MCO_3 + NO_2$	Equil. Constant / k12, JPL 19-5[6] page 521 Table 3-1

Note 1: Photolysis rate taken from the GEOS-CF[7] full chemical state sampled using the KPP Standalone Interface at a grid cell over Los Angeles at local noon[8]

Note 2: Termolecular reaction with effective second-order rate constant calculated from low pressure and high pressure limits and total gas concentration[6]

Table S3. Initialization of active species concentrations		
Name	Symbol	Range
Ozone	O_3	0 - 50 ppb
Nitric oxide	NO	0 - 20 ppb
Nitrogen dioxide	NO_2	0 - 20 ppb
Formaldehyde	HCHO	0 - 50 ppb
Hydroperoxyl radical	HO_2	0 – 0.01 ppb
Hydrogen peroxide	HO_2H	0 - 10 ppb
Hydroxyl radical	OH	0 – 0.001 ppb
Peroxyacetyl radical	MCO_3	0 – 0.01 ppb
Peroxyacetyl nitrate	PAN	0 - 25 ppb
Acetaldehyde	ALD2	0 – 10 ppb
Methylglyoxal	MGLY	0 - 50 ppb

The table above details the ranges for species concentrations, which are randomly initialized linearly within these ranges at the beginning of each hour of simulation. All active species have ranges except for diatomic oxygen which is initialized to a mixing ratio of 21%. Unlike previous work using this model, no pseudo steady-state approximation is used for the OH radical. Build-up species concentrations are initialized at zero, including water, neglecting effects of relative humidity in this mechanism beyond photolytic rates in a surface grid cell containing Los Angeles obtained from a GEOS-CF 100km simulation. For the scope of the Southern California surface grid cell midday simulation, pressure and temperature are fixed at 298 K and 1 atm.

S4. Precision extent of conservation

Achieving perfect conservation is bounded by machine precision constraints. While the XGBoost does not have any built-in constraints, the other 3 methods are ostensibly conservative. However, the 3 conservative methods differ in extent of conservation from the perspective of precision (though we note that the Rosenbrock solver and the projection methods are all acceptably conservative, as detailed in sections 2.4 and 3.1). We find that the correction with species-level weighting is the most numerically precise out of all predictions, from a distributional perspective. Figure S1 shows the distributions of atom imbalances similarly to Figure 3, but now using absolute tendencies on a log scale for all types of predictions (the reference model, XGBoost, unweighted correction and correction). The largest difference is between XGBoost and the other 3 ostensibly conservative methods. The nudging approaches conserve all atoms to higher precision than the original reference model using a Rosenbrock solver with an exact Jacobian. Though the nudging approaches are both very precise and have overlapping distributions, the weighted correction is visually shifted towards smaller deviations. On average, the deviations of the weighted correction are 2.5 times smaller than the unweighted correction.

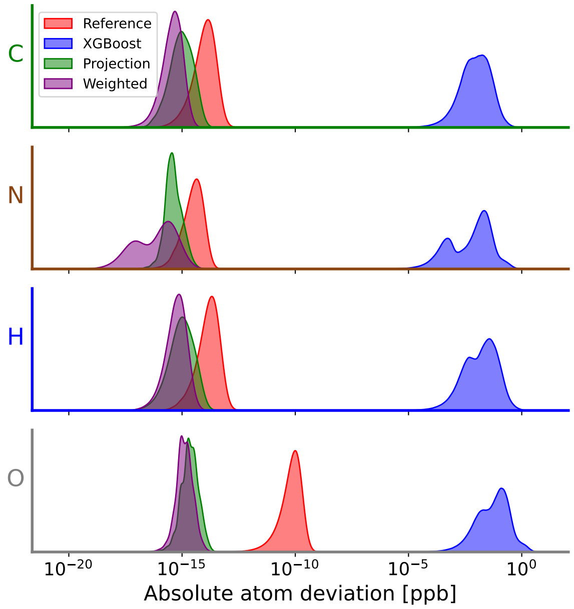

Figure S1. Distribution of atom imbalances from predictions of all 4 numerical methods. Distributions are created using kernel density estimates of the absolute atom balances.

The strict conservation of the weighted approach and its scale-aware adjustment motivate application to machine learning models as well as other numerical methods. While many numerical models make only small unphysical deviations, a small nudge can ensure that predictions are strictly conservative to very high precision.

S5. Scatter plots for all other species

This section contains scatter plots of accuracy for the predictions as in Section 3.2 in the main text, for all other species in the Julia photochemical mechanism.

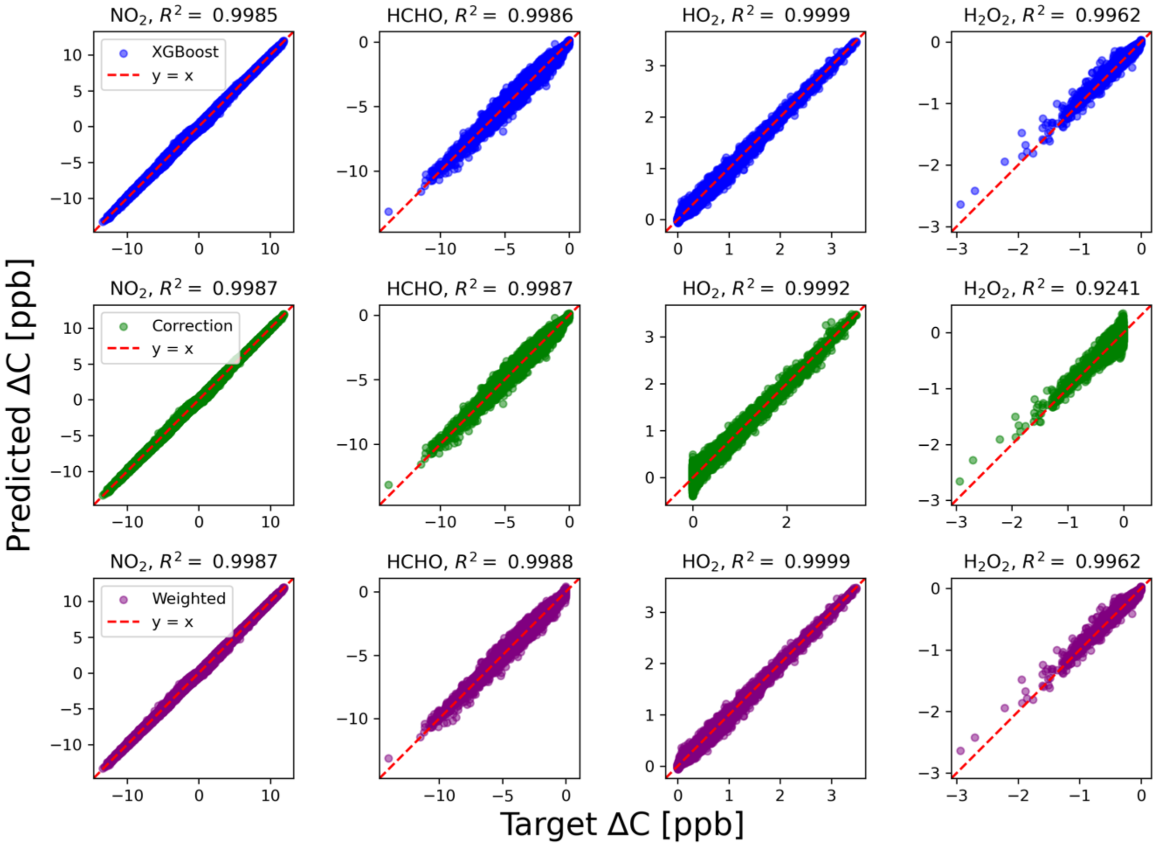

Figure S2. Scatter plot of NO_2, HCHO, HO_2, and H_2O_2 with the uncorrected predictions, corrected predictions and the weighted correction.

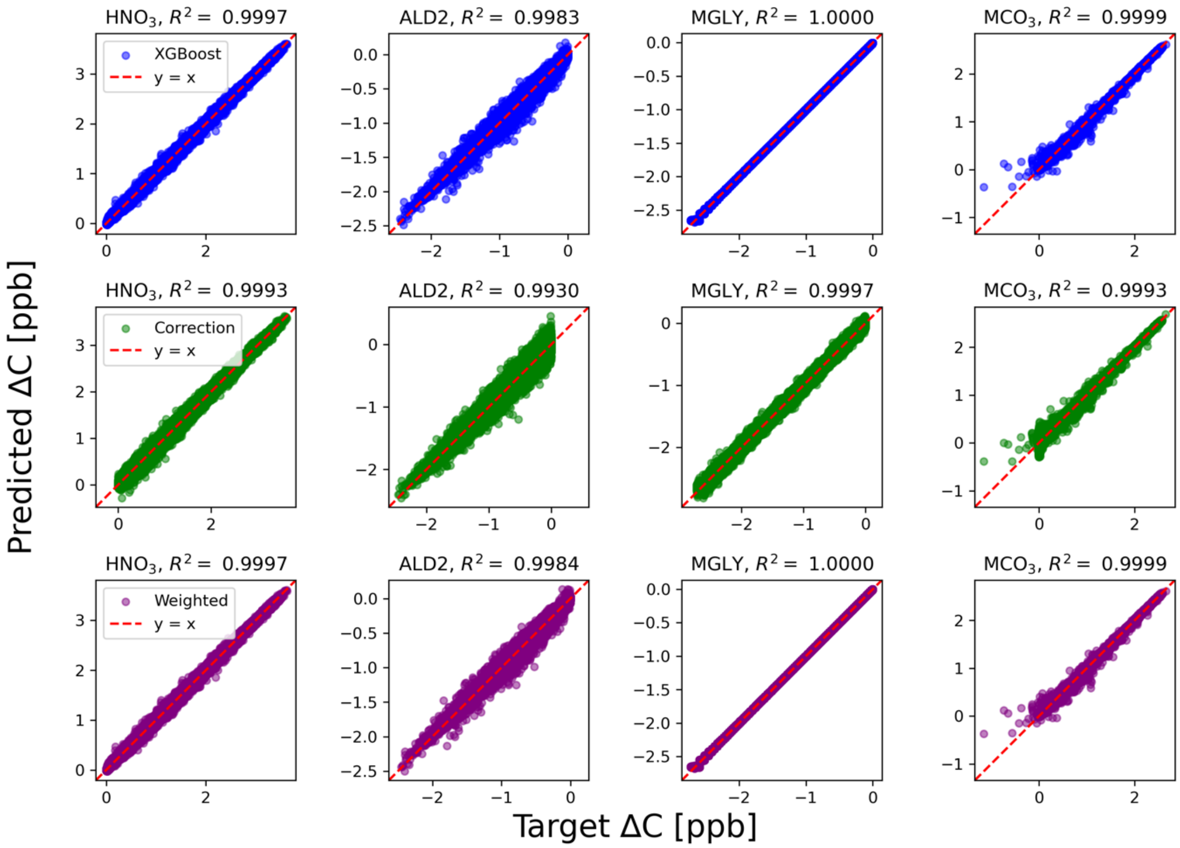

Figure S3. Scatter plot of HNO_3, ALD2, MGLY, and MCO_3 with the uncorrected predictions, corrected predictions and the weighted correction.

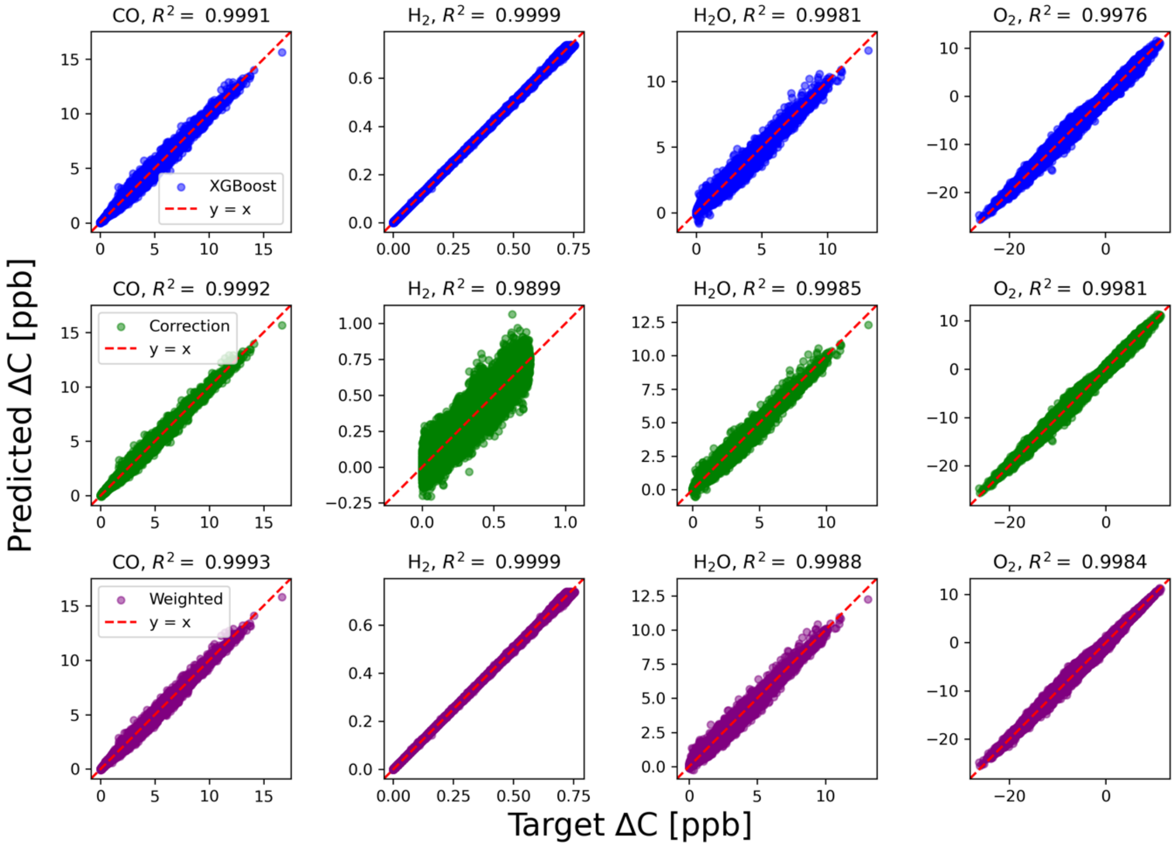

Figure S4. Scatter plot of CO, H_2, H_2O, and O_2 with the uncorrected predictions, corrected predictions and the weighted correction.

S6. Comparison to the flux-based approach for previously trained neural networks

We can demonstrate these methods on prior neural networks (NN) trained to emulate a prior version of the Julia photochemical model[9,10]. The version of the Julia photochemical model used here contained 11 species and 10 reactions. In this work, conservation of carbon and nitrogen atoms was the focus: oxygen atoms were not conserved, as diatomic oxygen was treated as an infinite source and sink, and hydrogen atoms were not conserved, as water was not tracked by the model. We demonstrate here that the nudging approach can be used to just conserve a subset of atoms.

This prior work compared a physics-constrained NN embedded with the mass-conserving framework to a more standard architecture "intermediate" complexity NN without physical constraints but with a similar number of trainable hyperparameters. We apply this post-prediction correction to the output of this intermediate NN. Figure S4 shows that this has only a minor effect on accuracy of 4 key species (ozone, NOx, and formaldehyde) relative to the prediction of the Julia photochemical model. For NO_2, R^2 value of tendencies even agrees more closely with the Julia photochemical model after the atom-conserving correction is applied to the intermediate NN, reaching 0.95, which is the same as the physics-constrained NN embedded with the graph locality of the chemical mechanism. Numerical tests over all test data show that total carbon is conserved in all cases within 10^{-17} ppb and total nitrogen within 10^{-15} ppb.

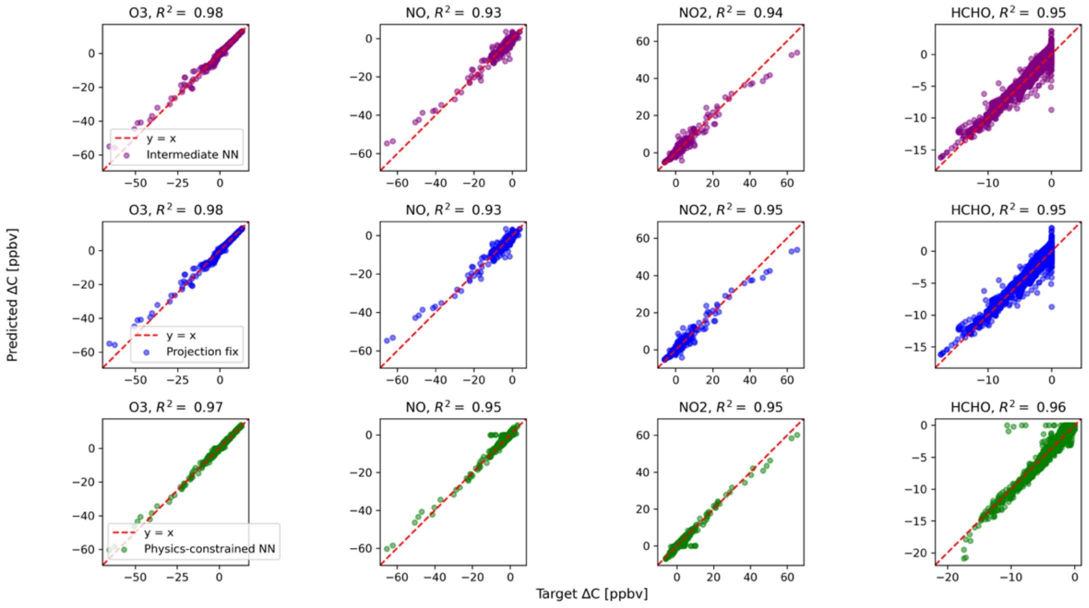

Figure S5. Scatter plot of target vs predicted tendencies, for the intermediate NN, the intermediate NN with the atom-conserving tendency correction using the methods from Section 2.1, and the physics-constrained NN from Sturm and Wexler[10].

References

(1) Boyd, S.; Vandenberghe, L. *Introduction to Applied Linear Algebra: Vectors, Matrices, and Least Squares*, 1st ed.; Cambridge University Press & Assessment, 2018. https://doi.org/10.1017/9781108583664.

(2) Kelp, M. M.; Jacob, D. J.; Kutz, J. N.; Marshall, J. D.; Tessum, C. W. Toward Stable, General Machine-Learned Models of the Atmospheric Chemical System. *Journal of Geophysical Research: Atmospheres* **2020**, *125* (23), e2020JD032759. https://doi.org/10.1029/2020JD032759.

(3) Kelp, M. M.; Jacob, D. J.; Lin, H.; Sulprizio, M. P. An Online-Learned Neural Network Chemical Solver for Stable Long-Term Global Simulations of Atmospheric Chemistry. *Journal of Advances in Modeling Earth Systems* **2022**, *14* (6), e2021MS002926. https://doi.org/10.1029/2021MS002926.

(4) Yang, X.; Guo, L.; Zheng, Z.; Riemer, N.; Tessum, C. W. Atmospheric Chemistry Surrogate Modeling With Sparse Identification of Nonlinear Dynamics. *Journal of Geophysical Research: Machine Learning and Computation* **2024**, *1* (2), e2024JH000132. https://doi.org/10.1029/2024JH000132.

(5) Sturm, P. O.; Manders, A.; Janssen, R.; Segers, A.; Wexler, A. S.; Lin, H. X. Advecting Superspecies: Efficiently Modeling Transport of Organic Aerosol With a Mass-Conserving Dimensionality Reduction Method. *Journal of Advances in Modeling Earth Systems* **2023**, *15* (3), e2022MS003235. https://doi.org/10.1029/2022MS003235.

(6) Burkholder, J. B.; Sander, S.P.; Abbat, J.; Barker, J.R.; Cappa, C.; Crounse, J.D.; Dibble, T.S.; Huie, R.E.; Kolb, C.E.; Kurylo, M.J.; Orkin, V.L.; Percival, C.J.; Wilmouth, D.M.; Wine, P.H. JPL Publication 19-5. Chemical Kinetics and Photochemical Data for Use in Atmospheric Studies. **2020**.

(7) Keller, C. A.; Knowland, K. E.; Duncan, B. N.; Liu, J.; Anderson, D. C.; Das, S.; Lucchesi, R. A.; Lundgren, E. W.; Nicely, J. M.; Nielsen, E.; Ott, L. E.; Saunders, E.; Strode, S. A.; Wales, P. A.; Jacob, D. J.; Pawson, S. Description of the NASA GEOS Composition Forecast Modeling System GEOS-CF v1.0. *Journal of Advances in Modeling Earth Systems* **2021**, *13* (4), e2020MS002413. https://doi.org/10.1029/2020MS002413.

(8) Li, E. W.; Sturm, P. O.; Silva, S. J.; Barber, V. A.; Keller, C. A. Characterizing the Speed of Chemical Cycling in the Atmosphere. July 11, 2024. https://doi.org/10.22541/essoar.172072964.41409656/v1.

(9) Sturm, P. O.; Wexler, A. S. A Mass- and Energy-Conserving Framework for Using Machine Learning to Speed Computations: A Photochemistry Example. *Geoscientific Model Development* **2020**, *13* (9), 4435–4442. https://doi.org/10.5194/gmd-13-4435-2020.

(10) Sturm, P. O.; Wexler, A. S. Conservation Laws in a Neural Network Architecture: Enforcing the Atom Balance of a Julia-Based Photochemical Model (v0.2.0). *Geoscientific Model Development* **2022**, *15* (8), 3417–3431. https://doi.org/10.5194/gmd-15-3417-2022.
